# Fuel shortages during hurricanes: Epidemiological modeling and optimal control

**DOI:** 10.1371/journal.pone.0229957

**Published:** 2020-04-01

**Authors:** Sabique Islam, Sirish Namilae, Richard Prazenica, Dahai Liu

**Affiliations:** 1 Aerospace Engineering, Embry-Riddle Aeronautical University, Daytona Beach, Florida, United States of America; 2 Department of Graduate Studies, Embry-Riddle Aeronautical University, Daytona Beach, Florida, United States of America; Shandong University of Science and Technology, CHINA

## Abstract

Hurricanes are powerful agents of destruction with significant socioeconomic impacts. A persistent problem due to the large-scale evacuations during hurricanes in the southeastern United States is the fuel shortages during the evacuation. Computational models can aid in emergency preparedness and help mitigate the impacts of hurricanes. In this paper, we model the hurricane fuel shortages using the SIR epidemic model. We utilize the crowd-sourced data corresponding to Hurricane Irma and Florence to parametrize the model. An estimation technique based on Unscented Kalman filter (UKF) is employed to evaluate the SIR dynamic parameters. Finally, an optimal control approach for refueling based on a vaccination analogue is presented to effectively reduce the fuel shortages under a resource constraint. We find the basic reproduction number corresponding to fuel shortages in Miami during Hurricane Irma to be 3.98. Using the control model we estimated the level of intervention needed to mitigate the fuel-shortage epidemic. For example, our results indicate that for Naples- Fort Myers affected by Hurricane Irma, a per capita refueling rate of 0.1 for 2.2 days would have reduced the peak fuel shortage from 55% to 48% and a refueling rate of 0.75 for half a day before landfall would have reduced to 37%.

## Introduction

Hurricanes are a periodic socio-economic threat for population centers in coastal areas globally. There is evidence for increased hurricane activity in the industrial era [1], and a rise in the number of high-intensity hurricanes over the past four decades [2]. Hurricanes have a severe socio-economic effect over extended geographic areas and impact the health and safety of residents in coastal regions like Florida. Computational modeling integrated with new social media data sources can assist in emergency preparation and evacuation efforts which save lives.

In the past decade, hurricanes impacting the Southeastern United States have led to high volume evacuations. The 2017 evacuation from Hurricane Irma has been referred to as the largest evacuation in the history of the nation. During this hurricane, twenty-three counties in Florida issued mandatory evacuation orders, and the remaining forty-four counties placed voluntary orders. Analysis of Hurricane Irma traffic data obtained from the Florida Department of Transportation (FDOT) indicates a net exodus of 550,000 vehicles from the southern parts of Florida. It is estimated that approximately 6.8 million Floridians and tourists took to the roads in the days leading up to the storm [3]. Such mass evacuations have also been observed during Hurricane Florence [4], affecting North and South Carolinas, as well as during Hurricane Michael [5]. Hurricane evacuees tend to make longer, intercity trips to stay with friends and family outside the impacted area and to completely move out of the storm path [6].

The high-volume mass evacuations, disruptions to the supply chain, long distances traveled, and fuel hoarding from non-evacuees have led to localized fuel shortages lasting several days and a cascade of problems in hurricane-affected areas. For example, evacuation during Hurricane Irma created a widespread fuel shortage problems days before the hurricane’s landfall for most of Florida and especially for South Florida. The fuel shortage problems gave rise to various other issues such as an unpredictable increase in fuel prices that exasperate and hinder evacuees living in low-income areas, traffic congestion on the highways due to stranded vehicles, and difficulties with emergency and medical transportation needs [3]. Understanding the characteristics of fuel shortage during hurricane evacuation is crucial to the mitigation of this problem and reducing the casualties caused by an imminent hurricane. The data explosion from social media enables new analysis approaches for this problem. For example, a recent study examines twitter data to predict fuel shortages during disasters [7].

While news reports have documented fuel shortages during the past hurricanes, crowd-sourced data from the social media platform Gasbuddy [8] has quantified the shortages during recent hurricanes. The progression of fuel shortage through a geographic area and the return to normal fuel supply has similarities with the spread of infectious diseases. For example, a refueling station in the vicinity of another station that is out of gas is more likely to be depleted of fuel soon, similar to infectious disease spread. Sociologists and computational scientists have long studied social events using biological models of infectious disease spread. Modeling interconnected social events as contagion leads to the analysis of these events in a new light. For example, a recent study by Towers et al [9] utilized epidemic modeling to examine mass killings related to gun violence and found that the likelihood of a mass killing increased because of a preceding occurrence of a similar event. Contagious disease modeling has been used to study several social phenomena that show epidemic like behavior such as: election campaign donations [10], spread of emotional influence in social media [11], suicidal ideation [12], spread of web malware [13], social contagion of altruism [14], etc. Recent studies have combined the biological and social contagious behaviors, e.g. Fu and coworkers [15] studied the interaction between the spread of the influenza infection, and the corresponding social media trends about flu-vaccine. These studies point to the success of epidemiological models in examining the dynamics of problems involving social contagion.

**Fig 1:**
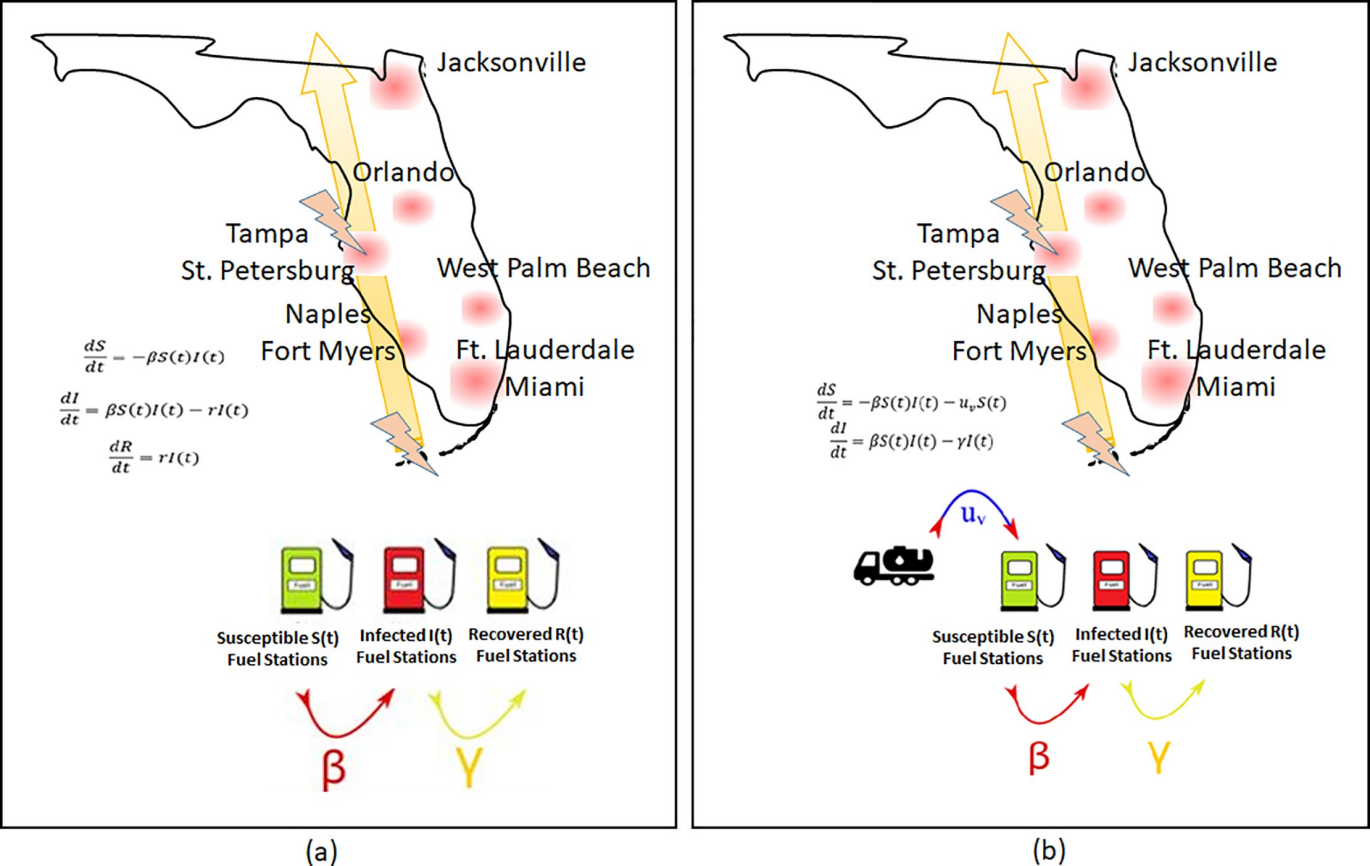
(a). SIR dynamics model repurposed to study fuel shortages during hurricane evacuation (b) SIR dynamics model augmented to include vaccination rate as per capita rate of refueling, u_v_.

The well-studied classical compartmental epidemic models used in most of the above studies such as SIS (Susceptible-Infected-Susceptible), SIR (Susceptible-Infected-Recovered) and SIRS (Susceptible-Infected-Recovered-Susceptible) divide the host population into susceptible, infected and recovered compartments with a set of differential equations describing dynamics between these different compartments [16]. In this study, we apply the SIR dynamics to model fuel shortage during hurricane evacuation as an epidemic and examine the infection dynamics as shown in the schematic in Fig 1. We further apply optimal control theory to determine an optimal refueling strategy utilizing an SIR with vaccination analogue to estimate the refueling needs to mitigate the epidemic, subject to resource constraints.

We utilize the data from the crowd sourced platform Gasbuddy for Hurricane Irma to parametrize the model. The unique advantage of this data source is the easy access to the on-the-fly data as the evacuation and fuel shortages are evolving during a hurricane. While the Gasbuddy fuel shortage data exhibit the characteristics of an epidemic, the optimal refueling model is based on a time invariant continuous SIR model represented by continuous ordinary differential equations [17, 18]. To address this problem, we use the Unscented Kalman Filter (UKF) algorithm to numerically estimate the SIR model parameters that characterize the dynamics as a continuous SIR model, while closely resembling the fuel shortage empirical data. To the best of our knowledge, this is the first application of epidemiological modeling and optimal control algorithms to the problem of fuel shortages during hurricanes. The mathematical development for the problem is presented first, followed by the results and discussions.

## Methods

### Data sources

The fuel shortage data for this study were obtained from Gasbuddy news releases during evacuation due to Hurricanes Irma and Florence. Gasbuddy is an online database containing vital roadside information on more than 150,000 fuel stations [8]. Gasbuddy played a crucial role during Hurricanes Irma and Florence by connecting evacuees and providing real-time information on fuel availability in the affected areas during the evacuation. A recent article in The Wall Street Journal reported that the Gasbuddy Mobile app was downloaded 300,000 times during the events leading up to Hurricane Irma, compared to 30,000 times on a typical day [19]. One problem with crowd sourced data is the reliability of the data. Gasbuddy cross-checks the reported data with the user’s location information to improve reliability [19]. Levin et al [20] report that reliability of volunteer generated data is improved by using multiple sources.

Hurricane Irma made landfall near Cudjoe Key, on September 10th, 2017 at 9:00 AM ET. Another landfall occurred on September 10^th^ 3:35 PM at Marco Island near Naples [21]. This led to large scale evacuation of affected areas in the preceding days. Gasbuddy provides continuous updates of aggregate fuel shortage levels in various affected cities on their website during an ongoing hurricane evacuation [8]. For example, Gasbuddy reported the data about the percentage of refueling stations out of fuel in major cities in Florida including Fort Myers- Naples, Miami-Fort Lauderdale, Tampa-St Petersburg, Orlando and Jacksonville from 9/6/2017 to 9/18/2017. Hurricane Florence, a slow-moving storm, damaged several regions in North and South Carolina in September 2018 and resulted in fuel shortages as high as 70% in some cities like Wilmington, North Carolina. The aggregate data obtained from Gasbuddy website corresponding to these hurricanes was used to parametrize our model. In addition, we use traffic data from the Florida Department of Transportation (FDOT) [22] and demographic data from the United States Census Bureau [23] in this work.

### SIR dynamics for fuel shortages

In the SIR model, schematically shown in Fig 1(A), we treat the percentage of refueling stations without gasoline as “infected (I)”, percentage of refueling stations with gasoline that are prone to running out of gasoline as “susceptible (S)” and percentage filled with gasoline after running out of fuel as “recovered (R)”. The recovered refueling stations do not get re-infected (experience fuel shortage) in this case as the model and the on-ground situation represents a short-term outbreak. In terms of differential equations, the dynamic model for the SIR is:

$$\frac{dS}{dt}=-\beta S(t)I(t)$$ Eq 1

$$\frac{dI}{dt}=\beta S(t)I(t)-\gamma I(t)$$ Eq 2

$$\frac{dR}{dt}=\gamma I(t)$$ Eq 3

The parameters β and γ represent the transmission rate per capita and recovery rate, which in the current context represent the rate at which the susceptible refueling stations are emptying and the empty gas stations are resupplied respectively. The quantity *βS*(0)/*γ* is a threshold quantity known as a basic reproduction number (*R*_0_). Here, we define it as the % of refueling stations without fuel in a region, because of 1% stations going out of fuel.

We use the crowdsourced data from the Gasbuddy website in conjunction with the Unscented Kalman Filter (UKF) to estimate the *β* and *γ* parameters. The Kalman Filter [24, 25], developed in the early 1960’s, is an effective technique designed to estimate the parameters with measurement correction from empirical data. One of the earliest usages of the Kalman Filter was in the Apollo program [26], and it has seen widespread use in applications such as spacecraft reentry [27] and autonomous navigation through obstacle environments [28], as well as a diverse array of other engineering and epidemiological applications [29–31]. The use of the Kalman Filter for this parameter estimation problem, as opposed to conventional curve fitting techniques, facilitates bounding of the dynamic parameters. Furthermore, the Kalman Filter provides a convenient framework for extending the current parameter estimation approach to also address state estimation for dynamical systems. In this paper, the Kalman Filter is used to estimate the best fit constant values for the parameters β and γ, which are in turn used to develop the time invariant continuous SIR model that most closely resembles the empirical data. Different variations of Kalman Filter algorithms, such as the Extended Kalman Filter (EKF) [32], Ensemble Kalman Filter (EnKF) [33, 34], and Sigma Point or Unscented Kalman Filter (UKF) [35], have been developed and used for various applications in engineering and epidemiology [24–35].

While the classical Kalman filter provides optimal state and parameter estimation for linear systems subject to Gaussian white noise, the process equations for the SIR problem, shown in Eqs 4 and 5, are inherently nonlinear, and the process and measurement noise are not necessarily Gaussian. The EKF algorithm can address nonlinearity by using the nonlinear dynamics for state propagation along with linearized dynamics to propagate the error covariance. The Sigma Point or Unscented Kalman Filter (UKF) can accommodate nonlinear dynamics without being constrained by the limitations associated with linearized models, which can be a poor approximation of highly nonlinear systems or processes. Instead, the UKF characterizes the estimation error by propagating a set of sigma points through the nonlinear dynamics model. Other approaches have been developed that do not require the assumption of Gaussian noise distributions. For example, the particle filter [36], also known as sequential Monte Carlo analysis [37], propagates a large number of random samples (or particles) in an effort to capture the noise distribution, but this approach entails significant computational cost. Therefore, the UKF is used in this work for the estimation of the SIR model parameters as it typically provides superior performance than the EKF at a similar cost without the intense computational burden associated with a particle filter. The estimated model parameters are then used in the optimal control algorithm to estimate an optimal refueling strategy.

By employing the fuel shortage data from Gasbuddy for the measurement update, we can simultaneously generate the synthetic data for the mechanistic SIR Model and estimate the parameters *β* and *γ*. The differential equations of the Fuel Shortage SIR model are then converted to discrete time form at different days, k, using the Euler Method. The state vector that is input into the UKF is defined as *X*_*k*_ = [*S*_*k*_,*I*_*k*_,*β*_*k*_,*γ*_*k*_]^*T*^.

That is, the states are susceptible, infected, and recovered refueling stations and the parameters β and γ are the rates at which susceptible refueling stations are infected and infected refueling stations are recovered. The process equations, using the Euler method, for the UKF are then setup as shown below:

$$S_{k}=S_{k-1}+(-\beta_{k-1}S_{k-1}I_{k-1})dt$$ Eq 4

$$I_{k}=I_{k-1}+(\beta_{k-1}S_{k-1}I_{k-1}-\gamma_{k-1}I_{k-1})dt$$ Eq 5

$$\beta_{k}=\beta_{k-1}$$ Eq 6

$$\gamma_{k}=\gamma_{k-1}$$ Eq 7

The output then takes the form of:

$$Y_{1,k}=S_{k}$$ Eq 8

$$Y_{2,k}=I_{k}$$ Eq 9

The Unscented Kalman Filter relies on the unscented transformation, which determines the statistics of an *L* dimensional random variable *x* through a nonlinear transformation *y = f(x)*. It is assumed that the state vector *x* has a known initial mean $\bar{x}$ and initial covariance *P*_0_. The main goal of the UKF is to reduce the error in state estimation from a priori *(k-1)* value to a posteriori *(k)* value in each successive time interval *dt* for *N* time steps. For Hurricane Irma, the entire time interval is 12 days with a time step of 0.25 days. For Hurricane Florence, more refined data were available, so we used a time step of 1 hour for the interval of 18 days. The statistics of the function *y* can then be determined using the procedures listed in the UKF pseudo-code as shown in Table 1.

**Table 1:** Unscented Kalman Filter estimation process.

Step	Equation	Comment
**1. Initialization**	$X_{0}=E[X_{k=1}],\;{\hat{X}}_{0}=E[{\hat{X}}_{k=1}]$, $P_{0}=E\left[(X_{k=1}-{\hat{X}}_{k=1}){\left(X_{k=1}-{\hat{X}}_{k=1}\right)}^{T}\right]$ *Q* = *L* x *L* process noise covariance matrix *R* = *p* x *p* measurement noise covariance matrix ${\left(P_{k}^{yy}\right)}_{i=1}=R$ ${\left(P_{k}^{xy}\right)}_{i=1}={\left[0\right]}_{L\times 2}$	k = 1,2…, N. N = dimension of time interval divided in dt steps. p = number of outputs in Y_k_ i = 1,2…, 2L+1. L = number of states in X_k_.
**2. Define Scaling Factor and Compute Weighting Matrices**	**Scaling Factors** *α*,*β* and *κ* are constant scaling factors *λ* = *α*^2^(*L*+*κ*)−*L*, where *L* = *size of X*_*k*_ **Weighting Matrix** $W_{m}^{1}=\frac{\lambda }{L+\lambda }$ $W_{c}^{1}=\frac{\lambda }{L+\lambda }+(1-\alpha^{2}+\beta ),$ $W_{m}^{j}=W_{c}^{j}=\frac{1}{\left[2(L+\lambda )\right]}\;for\;j=2,\ldots .,2L+1$	In the current implementation *α =* 1,*β* = 2,*κ* = 0 Assume Gaussian weighting distribution
**3. Generation of Sigma Points**	$\sqrt{P_{k}}=Chol(P_{k})$ $\chi_{k-1}=[{\hat{X}}_{k-1}\;{\hat{X}}_{k-1}[1\;1\;1\;1]+\sqrt{(L+\lambda )P_{k-1}}$ ${\hat{X}}_{k-1}[1\;1\;1\;1]-\sqrt{(L+\lambda )P_{k-1}}]$ Use the nonlinear system equations (Eqs 4–7) to propagate the sigma points: $\chi_{k|k-1}^{i}=f(\chi_{k-1}^{i})\;for\;i=1,2,\ldots .,2L+1$	Chol represents the Cholesky Decomposition
**4. Compute Mean and Error Covariance**	${\hat{X}}_{k|k-1}=\sum_{i=1}^{2L+1}W_{m}^{i}\chi_{k|k-1}^{i}$ $P_{k|k-1}=Q+\sum_{i=1}^{2L+1}W_{c}^{i}(\chi_{k|k-1}^{i}-{\hat{X}}_{k|k-1}){\left(\chi_{k|k-1}^{i}-{\hat{X}}_{k|k-1}\right)}^{T}$	${\hat{X}}_{k|k-1}$ mean of predicted state
**5. Generate Observations**	$\psi_{k|k-1}^{i}=h(\chi_{k|k-1}^{i})$ ${\hat{Y}}_{k|k-1}=\sum_{i=1}^{2L+1}W_{m}^{i}\psi_{k|k-1}^{i}$	${\hat{Y}}_{k|k-1}$ mean of predicted output
**6. Covariance and Cross Covariance Estimation**	$P_{k}^{yy}=\sum_{i=1}^{2L+1}W_{c}^{i}(\psi_{k|k-1}^{i}-{\hat{Y}}_{k|k-1}){\left(\psi_{k|k-1}^{i}-{\hat{Y}}_{k|k-1}\right)}^{T}$ $P_{k}^{xy}=\sum_{i=1}^{2L+1}W_{c}^{i}(\chi_{k|k-1}^{i}-{\hat{X}}_{k|k-1}){\left(\psi_{k|k-1}^{i}-{\hat{Y}}_{k|k-1}\right)}^{T}$	
**7. Compute Kalman Gain Matrix**	$K_{k}=P_{k}^{xy}{\left(P_{k}^{yy}\right)}^{-1}$	For updating state prediction & reducing estimation error.

In Step 1, we initialize the UKF by providing the initial values for state vector *X*_*k*_ for *t = 0* days (k = 1). We used a set of initial values of *S*_k_ and *I*_k_ from the Gasbuddy fuel shortage data for the first day. Initial values for β and γ were set to zero as they are to be determined through the estimation process. The initial covariance, P_0_ was set to the identity matrix with the same dimension as the state vector *X*_k_.

The Q and R are the process and measurement noise covariance matrices in the estimation and update steps (Step 3 to Step 8) shown in Table 1. In this implementation, Q and R were chosen as diagonal matrices with diagonal elements (10, 10, 100, 100) and (10, 10), respectively; these values were selected after some tuning of the UKF. The cross-covariance matrices $P_{k}^{yy}\;and\;P_{k}^{xy}$ were initialized to R and the identity matrices respectively. The model update step entails propagating a set of 2L+1(where L is the number of states) sigma points through the nonlinear dynamics model (Eqs 4–7). The mean of the state estimate (${\hat{X}}_{k|k-1}$) and the error covariance matrix (*P*_*k*|*k*−1_) are then updated as a weighted combination of the propagated sigma points as shown in Step 4. The weighting matrices are computed as shown in Step 2. The measurement update is performed by first generating a set of measurements ($\psi_{k|k-1}^{i}$) by propagating the sigma points through the output equation (Step 5). The current measurement (${\hat{Y}}_{k|k-1}$) is computed as a weighted combination of these propagated sigma points ($\psi_{k|k-1}^{i}$). The covariance and cross covariance estimation matrices are then updated as shown in Step 6, which in-turn are used to update the Kalman gain in Step 7. Finally, the mean of the state estimate and the error covariance matrix are updated in the last step. This process is then repeated until k = N. The states *S*_k_ and *I*_k_ are being updated at every time step, as are the states β and γ, defined by their relation to *S*_k_ and *I*_k_ in Eqs 4 and 5. In this process we can estimate the transmission rate (β) and recovery rate (γ) for every time step from the data provided by Gasbuddy.

### Optimal control algorithm for the refueling strategy

The Unscented Kalman Filter provides estimates of the parameters β and γ, which are constant scalar values that can be used to develop a continuous time invariant dynamic model to characterize the fuel shortage as an infection. We now utilize this dynamic model to determine an optimal refueling strategy, which is modeled like a vaccination intervention, to mitigate the hurricane fuel shortage. The resulting control law is a bang-bang control policy. Bang-bang controllers [38] typically arise in minimum-time problems with constrained inputs, such as spacecraft maneuvers using thruster control [39, 40]. The result is a control input that corresponds to the maximum or minimum value with a finite number of switching times. Hansen and Day [17] and Kang et al. [18] have shown, using SIR dynamic models, that the optimal vaccination policy for mitigating disease epidemics is a bang-bang policy. In this work, we employ a similar mathematical formulation for the gas shortage problem; leveraging theoretical results from [17] and [18], the optimal control policy in this case also corresponds to a bang-bang policy.

The SIR dynamics model is augmented to include vaccination [18] as shown below:

$$\frac{dS}{dt}=-\beta S(t)I(t)-u_{v}S(t)$$ Eq 10

$$\frac{dI}{dt}=\beta S(t)I(t)-\gamma I(t)$$ Eq 11

The term *u*_*v*_ in *Eq 10* is the per-capita rate of refueling. Keeping congruency with our model parameters, *u*_*v*_ is the rate at which susceptible gas stations are prevented from being emptied out by external intervention in the form of additional fuel supply. The control variable *u*_*v*_ is bounded by practical constraints. The level of *u*_v_ that can be attained at any given time depends on the infrastructure that is in place to overcome fuel shortage problems such as the amount of gasoline in reserve in proximity to the area in question, the availability of transport vehicles etc. This resource constraint is addressed through the optimal control algorithm.

Let *R(t)* denote the total number of refueled fuel stations that were susceptible to becoming empty (infected) at time, *t*. The actual values of *S*, *I* and *R* will depend on the specific choice of the control *u*_*v*_. Then, if *r*_*max*_≥0 is fixed, *u*_*v*_ needs to be determined for the augmented SIR model in Eq 10 that minimizes the cost function *(J)* shown in Eq 12. Similar approaches have been used for the vaccination analogue for infectious disease modeling [18].

$$J=\int_{t0}^{T}\beta S(t)I(t)\;dt$$ Eq 12

subject to $S(t_{0})=S_{0},I(t_{0})=I_{0},I(T)=I_{min},r(T)=r_{max},u_{v}(t)\in [0,u_{v,max}]$ for all *t*∈[0,*T*]. In Eq 12, *I*_*min*_ is a threshold constant chosen to indicate the end of the fuel shortage problem at some arbitrary final time *T*.

This optimal problem can be solved by applying Pontryagin’s Maximum Principle (PMP) [41]. Consider the following general optimal control problem with isoperimetric constraints:

$$\min J=\phi (T,x(T))+\int_{t_{0}}^{T}L(t,x,u)\;dt$$ Eq 13

such that

$$\left\{ \begin{array}{c} \frac{dx}{dt}=f(t,x,u),x(t_{0})=x_{0},u\in U,\\ \int_{t_{0}}^{T}L(t,x,u)dt=\int_{t0}^{T}\beta S(t)I(t)\;dt\;(Integral\;Cost\;Function)\\ Subject\;to\;the\;constraints:\\ R=\int_{t_{0}}^{T}u_{v}S(t)dt\leq r_{max}\;(Resource\;Constraint)\\ \psi (T,x(T))=0\;(Terminal\;Constraints)\\ {\left\{\phi_{x}+{\left[\psi_{x}\right]}^{T}\vartheta -\lambda (T)\right\}}^{T}{|}_{T}\;dx(T)+\{\phi_{T}+\vartheta^{T}\psi_{T}+H\}{|}_{T}\;dT=0\\ \left(Transversality\;Conditions\right) \end{array}\right.$$ Eq 14

where $x\in R^{n}$ is the state vector, *u* is the control input, *ψ* is a vector of terminal constraint functions, and *ϕ*, *L* are scalar-valued cost functions. U is an admissible control region, with continuous partial derivatives with respect to all its arguments [18].

From the optimal control problem in Eq 14, the Pontryagin Maximum Principle (PMP) states: if u*(t) is an optimal control with x*(t) being the optimal trajectory, there exists a non-trivial solution of vector functions *λ* (costate functions) and non-trivial constants *λ*_1_,*λ*_2_ *and ϑ* such that the conditions discussed below are satisfied:

$$\left\{ \begin{array}{c} \frac{dx}{dt}=f(t,x,u),\\ \frac{d\lambda }{dt}=-H_{x}^{T}(t,x,u,\lambda )\\ H(t,x,u^{*},\lambda ,\lambda_{1},\lambda_{2})\geq H(t,x,u,\lambda ,\lambda_{1},\lambda_{2})\forall \;admissible\;u\\ x(t_{0})=x_{0},\;\psi (T,x(T))=0,\\ H(T,x(T),u(T),\lambda (T),\lambda_{1}(T),\lambda_{2}(T))=-{\left[G_{T}^{T}(T,x(T),\vartheta )\right]}^{T}\\ \lambda (T)=G_{x(T)}^{T}(T,x(T),\vartheta )\\ L(t,x,u)dt=\int_{t0}^{T}\beta S(t)I(t)\;dt\\ \int_{t_{0}}^{T}R(t,x,u)dt\leq r_{max}\\ \lambda_{2}(\int_{t_{0}}^{T}R(t,x,u)dt-r_{max})=0,\;\lambda_{2}\geq 0, \end{array}\right.$$ Eq 15

where $H(t,x,u,\lambda ,\lambda_{1},\lambda_{2})=\lambda_{1}L(t,x,u)+\lambda^{T}(t)f(t,x,u)+\lambda_{2}R(t,x,u)$ is the Hamiltonian, and *G*(*T*,*x*(*T*)) = *ϕ*(*T*,*x*(*T*))+*ϑ*^*T*^*ψ*(*T*,*x*(*T*)).

If the system in Eq 15 is time-invariant, the Hamiltonian, *H*, is constant [17] such that: *H*(*t*,*x*,*u*,*λ*,*λ*_1_,*λ*_2_) = *const*,*∀t*∈[*t*_0_,*T*].

### Optimal refueling strategy

The deterministic SIR model for refueling with limited resources can be modeled by the governing equations shown in Eq 11 with the addition of the resource constraint derivative:

$$\frac{dR}{dt}=u_{v}S$$ Eq 16

If we construct this problem as a maximization problem, then Pontryagin’s Maximum Principle (PMP) can be used to develop the relationship:

$$H(t)=-\lambda_{1}\beta SI-\lambda_{S}\beta SI-\lambda_{S}u_{v}S+\lambda_{I}\beta SI-\lambda_{I}\gamma I+\lambda_{r}u_{v}S$$ Eq 17

where the costate equations are satisfied as follows:

$$\begin{array}{c} \frac{d\lambda_{S}}{dt}=-(\lambda_{I}-\lambda_{1}-\lambda_{S})\beta I-(\lambda_{r}-\lambda_{S})u_{v}\\ \frac{d\lambda_{I}}{dt}=-(\lambda_{I}-\lambda_{1}-\lambda_{S})\beta I+\lambda_{I}\gamma \\ \frac{d\lambda_{r}}{dt}=0 \end{array}$$ Eq 18

For this specific problem, there is no terminal cost or terminal constraints. Hence, the transversality conditions can be reduced to:

$${\underline{\lambda }}^{T}(T)d\underline{x}(T)+H(T)dT=0$$ Eq 19

where $d\underline{x}(T)={\left[0,dS(T),0,0\right]}^{T}$ and ${\underline{\lambda }}^{T}(T)=[\lambda (T),0,\lambda_{I}(T),\lambda_{r}(T)]$, as *I(T)* and *R(T)* are fixed (constant) but *S(T)* is variable. Applying the PMP to the system in Eq 15:

$$\begin{array}{c} H(t,x,u_{v}^{*},\lambda ,\lambda_{1},\lambda_{2})\geq H(t,x,u_{v},\lambda ,\lambda_{1},\lambda_{2}),\forall \;admissible\;u_{v}\\ -\lambda_{S}u_{v}^{*}S+\lambda_{r}u_{v}^{*}S\geq -\lambda_{S}u_{v}S+\lambda_{r}u_{v}S\\ u_{v}^{*}S(\lambda_{r}-\lambda_{S})\geq u_{v}S(\lambda_{r}-\lambda_{S}) \end{array}$$ Eq 20

The optimal control then becomes bang-bang control [18] where the switching function is given by (*λ*_*r*_−*λ*_*S*_ = 0) and satisfies

$$u_{v}^{*}=\left\{ \begin{array}{l} u_{v,max},\;\lambda_{r}>\lambda_{S}\\ ?,\;\lambda_{r}=\lambda_{S}\\ 0,\;\lambda_{r}<\lambda_{S} \end{array}\right.$$ Eq 21

Following the development in Ref [18], it can be shown that that the optimal control is purely bang-bang, and there is no singular component or discontinuity. The ‘?’ in the Eq 21 indicates that the case where λ_r_ = λ_S_ represents a condition for which the control input is undefined; however, following [18], it will now be shown that this condition never occurs.

If *λ*_*r*_−*λ*_*S*_ on some interval *B*, then $\dot{\lambda_{S}}=0$ on *B*. Eq 18 then can be simplified to:

$$0=-(\lambda_{I}-\lambda_{1}-\lambda_{S})\beta I-(\lambda_{r}-\lambda_{S})u_{v}$$ Eq 22

Let *u*_*v*_ = 0; then *λ*_*I*_ = *λ*_1_+*λ*_*s*_. We can further postulate that $\dot{\lambda_{I}}=0$ on *B*. Hence, by Eqs 18 and 22, it must follow that *λ*_*I*_ = 0. Therefore, *λ*_*S*_ = −*λ*_1_ and then the only nonzero criteria for the variables on *B* is (*λ*,*λ*_*S*_,*λ*_*I*_,*λ*_*r*_) = (1,−1,0,−1). Furthermore, by Eqs 18 and 19, once $u_{v}^{*}$ becomes singular, it must remain singular throughout the whole interval *B*. This is the case since, *T*∈*B*,(*λ*,*λ*_*S*_,*λ*_*I*_,*λ*_*r*_) = (1,−1,0,−1) has to satisfy the transversality condition that *λ*_*S*_(*T*) = 0 shown in Eq 18. We can further postulate that, since the boundary condition posed by the transversality condition is not met, the optimal control is purely bang-bang control (i.e., the condition *λ*_*r*_ = *λ*_*S*_ never occurs and the control is well-defined at all times).

Now we examine the time at which the optimal control switches from 0 to *u*_*v*,*max*_. Denote the switching time as *t*_*s*_. The Hamiltonian, H, at switching time, *t*_*s*_ can be written as follows [18]:

$$H(t_{s})=-\frac{d\lambda_{I}{(t}_{s})}{dt}I(t_{s})=-\frac{d\lambda_{S}{(t}_{s})}{dt}S(t_{s})-\lambda_{I}(t_{s})\gamma I(t_{s})=0$$ Eq 23

Substituting $\dot{\lambda_{I}}(t_{s})=0$ into Eq 18 gives

$$\left(\lambda_{S}(t_{s})+\lambda_{1})\beta S(t_{s})=\lambda_{I}(t_{s})(\beta S(t_{s})-\gamma \right)$$ Eq 24

Considering the relations in Eqs 21 and 23, the pure bang-bang optimal control is defined:

$$\begin{array}{c} \lambda_{I}(t_{s})>0\;when\;\dot{\lambda_{S}}(t_{s})<0\to (0\to u_{v,max})\\ \lambda_{I}(t_{s})<0\;when\;\dot{\lambda_{S}}(t_{s})>0\to (u_{v,max}\to 0)\\ \lambda_{I}(t_{s})=0\;when\;\dot{\lambda_{S}}(t_{s})=0\to (no\;switch\;occurs) \end{array}$$ Eq 25

Since *λ*_*S*_(*t*_*s*_) = *λ*_*r*_ = *const*.,*λ*_*S*_(*t*_*s*_)+*λ*_1_ is either always positive, always negative or always zero. Suppose *λ*_*S*_(*t*_*s*_)+*λ*_1_ = 0. Then, by Eq 24, either *λ*_*I*_(*t*_*s*_) = 0 or $S(t_{s})=\frac{\gamma }{\beta }$. According to Eq 25, if *λ*_*I*_(*t*_*s*_) = 0, then no switching occurs.

Therefore, if $S(t_{s})=\frac{\gamma }{\beta }$ then the optimal control has only one switch and this switching occurs when *I*(t) is maximum, since *S*(t) is a monotonically decreasing function of time [18]. So, the possible control switches are:

$$u_{v}^{*}=\left\{ \begin{array}{l} u_{v,max},t\in [0,t_{s})\\ 0,\;t\in [t_{s},T] \end{array}\right.$$ Eq 26

We consider *λ*_*S*_(*t*_*s*_)+*λ*_1_>0. By using the relations derived in Eqs 24 and 25, it follows that:

$$\begin{array}{c} (i)\;\lambda_{I}(t_{s})>0\;and\;S(t_{s})>\frac{\gamma }{\beta }\;or\\ (ii)\;\lambda_{I}(t_{s})<0\;and\;S(t_{s})<\frac{\gamma }{\beta } \end{array}$$ Eq 27

Thus, by tracking the value of *S*(t) we can develop an algorithm to switch the control and determine the switching time analytically. In this SIR model for fuel shortage, the switching time, t_s_, refers to the time when one should supply extra fuel to the operating fuel stations (susceptible at time *t*), to keep them operational in order to optimally control the fuel shortage epidemic to favorable levels.

The term *u*_*v*_ is the vaccination control for the SIR dynamic system. In our model we treat *u*_*v*_ as the percentage of operational fuel stations, S(t), that is being replenished to avoid additional fuel stations to go out of fuel. This is different from the recovery rate (γ) which is the rate at which non-operational fuel stations, I(t), are being replenished to become operational again. The optimal refueling rate per capita, u_v_, is targeted at the susceptible compartment (S(t)) of the dynamic system. This has no effect on the recovery rate, γ.

The control is applied at u_v,max_ from t = 0 to a switching time, t_s_ to optimally reduce I(t), such that the basic reproduction number (R_0_) corresponding to the fuel shortage is less than 1, thereby mitigating the epidemic. The model suggests a combination of u_v,max_ and t_s_ to achieve this objective. This approach helps to introduce optimal refueling control earlier in the evacuation period before the hurricane landfall and can determine the extra amount of reserve fuel required and the time period in which refueling is most effective.

## Results and discussion

### Parameter estimation

**Fig 2:**
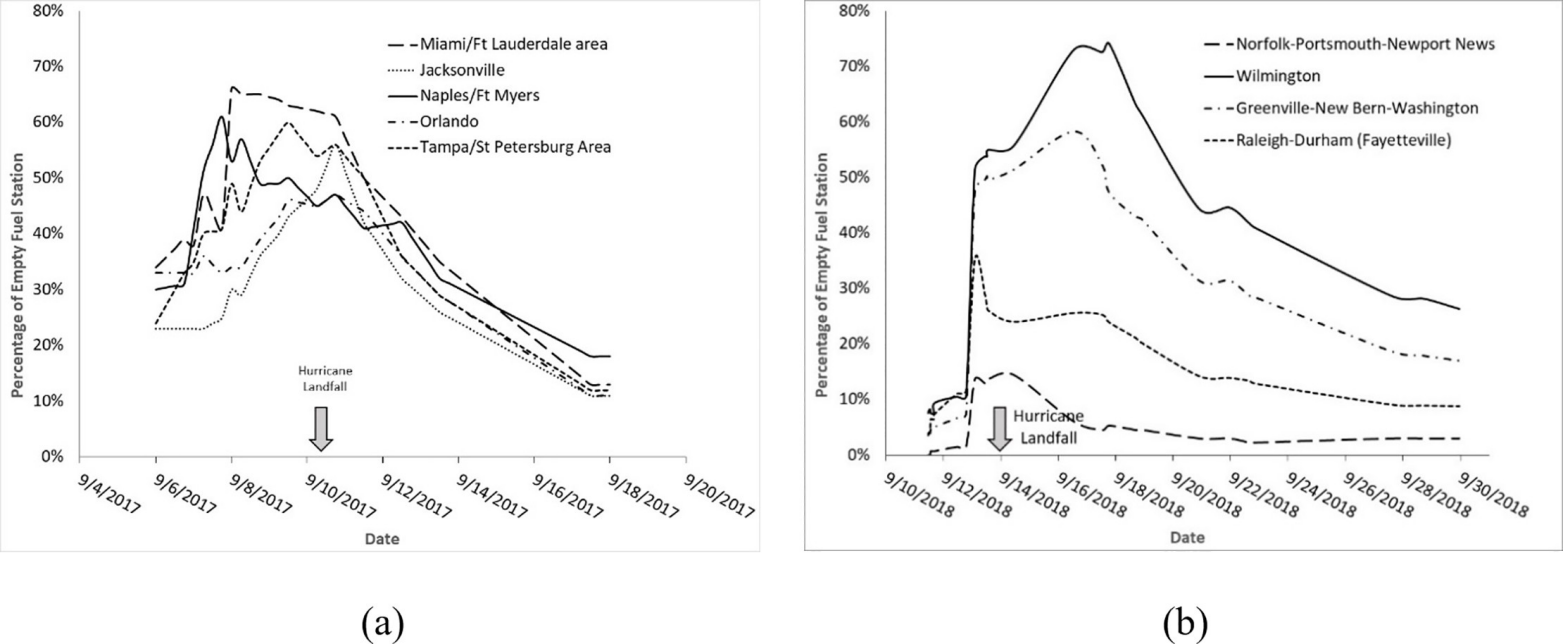
(a) The fuel shortage data from 2017 Hurricane Irma, (b) Similar data for 2018 Hurricane Florence.

The empirical data from the Gasbuddy crowdsourced platform are utilized to parameterize the models discussed above. The Unscented Kalman Filter is used to estimate the state variables and epidemic parameters (β, γ, R_0_) based on these data. Fig 2(A) shows the fuel shortage data for the 2017 Hurricane Irma and Fig 2(B) shows the similar data for the 2018 Hurricane Florence, which affected North Carolina. The plots indicate fuel shortages of up to 66% in South Florida during Hurricane Irma and similar shortages close to 70% in Wilmington, North Carolina during Hurricane Florence.

**Fig 3:**
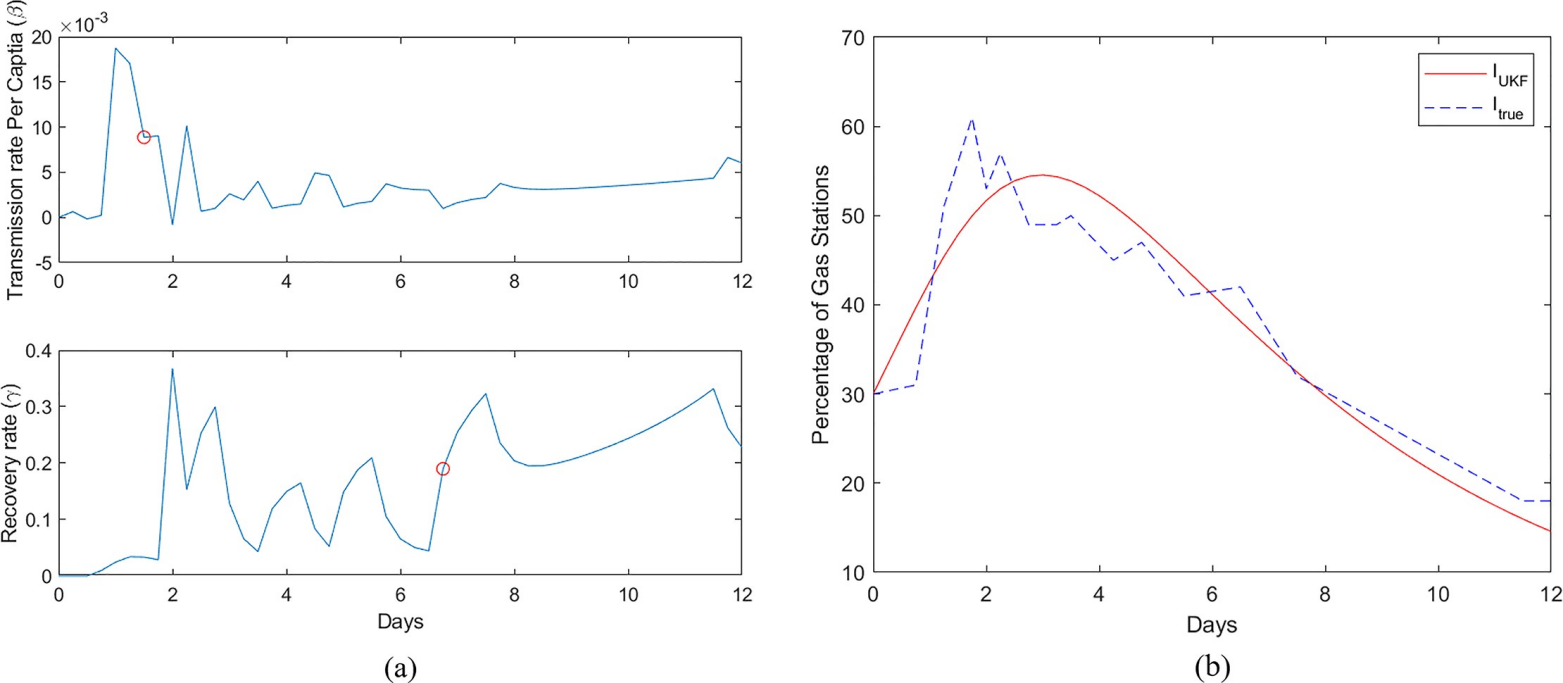
(a). β and γ rates estimated from Gasbuddy data for each time step (dt) for Fort Myers-Naples during Hurricane Irma. The red circle represents the β and γ values used to plot I_UKF_ in 3(b). (b) Continuous time Invariant data of % empty fuel stations (I(t)). Computed data from the best fit β and γ constant parameters, and the empirical data is shown for Fort Myers-Naples during Hurricane Irma.

**Fig 4:**
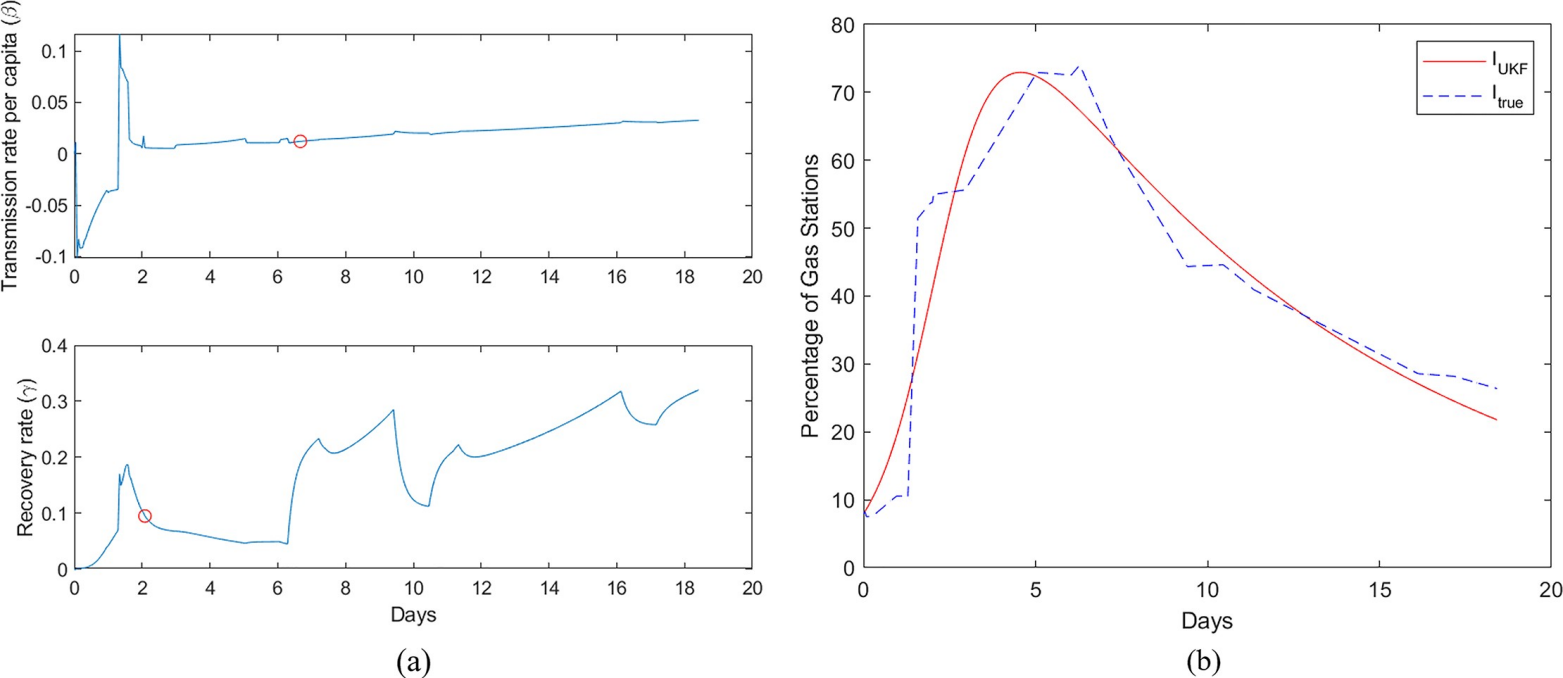
(a). β and γ rates estimated from Gasbuddy data for each time step (dt) for Wilmington during Hurricane Florence. The red circle represents the β and γ values used to plot I_UKF_ in 4(b). (b) Continuous time Invariant data of % empty fuel stations (I(t)). Computed data from the best fit β and γ constant parameters, and the empirical data is shown for Wilmington during Hurricane Irma.

Fig 3(A) shows the variation of transmission rate per capita (β) and the recovery rate (γ) estimated using UKF for the Fort Myers-Naples metropolitan area where Hurricane Irma had a landfall on continental United States. Similar data for Wilmington affected by Hurricane Florence is shown in Fig 4(A). While the fuel shortage data generally tend to peak ahead of the landfall in preparation for evacuation, the fluctuations in fuel demand observed in Fig 2 cause the variations in the parameter estimations for β and γ. Consider the variation of the β parameter; in both Figs 3(A) and 4(A) an initial peak is followed by a stabilization indicating the high demand for fuel as the evacuation is starting. Compared to the Fort Myers-Naples, Wilmington displayed higher values of β which is indicative of the fact that fuel shortages occurred at a faster rate in Wilmington during Hurricane Florence than in Fort Myers-Naples during Hurricane Irma. The γ rate shows a gradual increase after the hurricane is passed, indicating the progress of the recovery.

We require a constant parameter SIR dynamical system described by Eqs 1 and 2, for the implementation of the optimal control refueling strategy. For this purpose, the mechanistic data produced using all combinations of the β and γ values, estimated by the UKF were compared with the empirical data to evaluate the mean square error. The best fit β and γ values are marked in Figs 3(A) and 4(A) for the two cases. Fig 3(B) shows the empirical fuel shortage data and the estimated continuous data with the constant β and γ values for Naples-Fort Myers. A similar plot for Wilmington during Hurricane Florence is shown in Fig 4(B). In both cases, we can observe that the estimated data for the continuous time invariant SIR model show close resemblance to the empirical fuel shortage data.

**Fig 5:**
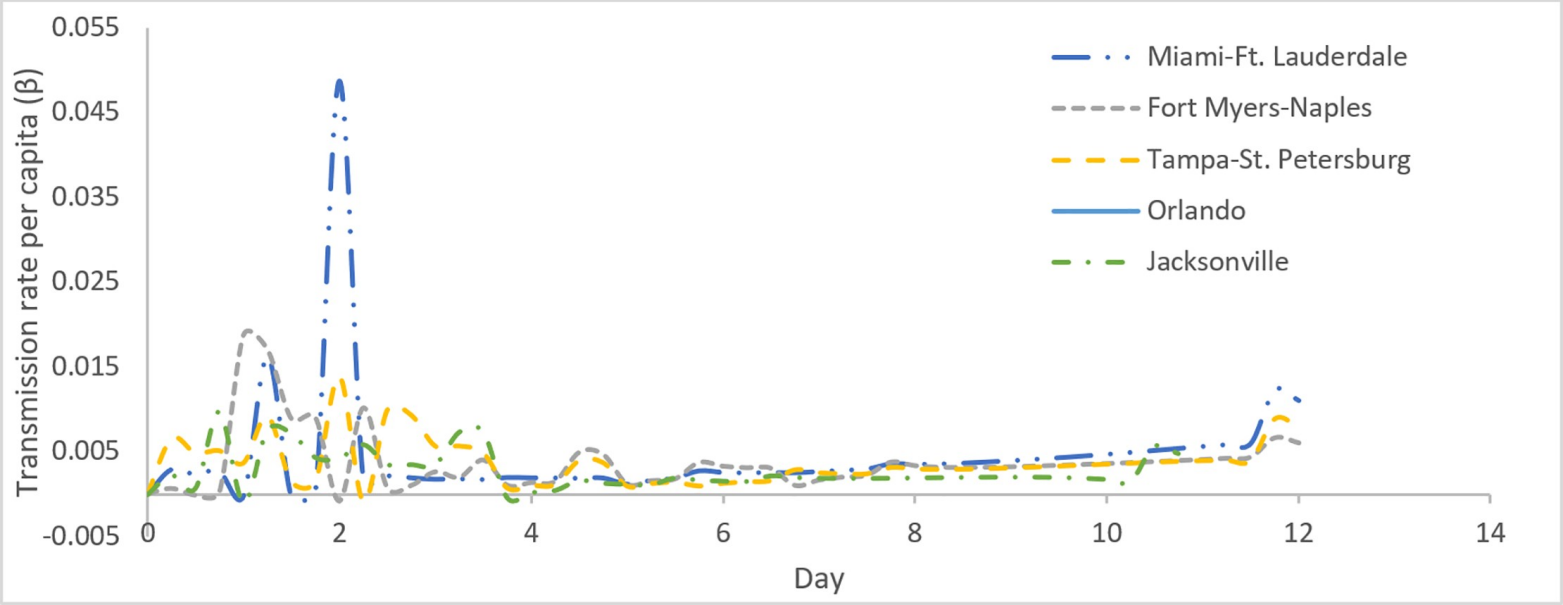
Transmission per capita rate (β) for the city/areas effected by Hurricane Irma.

The continuous time invariant model SIR data for the remaining cities affected by Hurricanes Irma and Florence were computed in a similar way. The best fit values of β, γ and the basic reproduction number (R0) for all of the cities are tabulated in Table 2. The UKF estimation of β and γ values were unique to each city. While the values of β vary depending on the impact of Hurricane evacuation in the different cities, the evolution of β follows a similar trend for all cities as shown in Fig 5. The similarity of γ values for different cities is indicative of the similarity in the recovery period for the different cities affected by Hurricane Irma. In the case of Hurricane Florence the slow moving nature of the storm and the difference in the infrastructure, in the affected communities resulted in the variation in the recovery periods.

The Unscented Kalman Filter used here is effective in reducing the linearization error when finding the parameters that generate data from Eqs 4 and 5 while representing the empirical data. In addition, this approach is effective in estimating the dynamic parameters with limited data during early stages of an ongoing hurricane evacuation. Such on-the-fly analysis can help decision makers allocate limited resources during an ongoing disaster.

**Table 2:** β, γ and R_0_ parameters and the number of fuel stations for the major cities affected by Hurricanes Irma and Florence.

Event	City/Area	Γ	β	R_0_	No. Of Fuel Stations
Irma	Miami-Fort Lauderdale	0.1841	0.0111	3.98	1369
	Fort Myers-Naples	0.1901	0.0089	2.90	76
	Tampa-St Petersburg	0.1708	0.01	3.40	922
	Orlando	0.2214	0.006	1.57	810
	Jacksonville	0.2718	0.0097	1.61	453
Florence	Wilmington	0.0953	0.012	11.59	46
	Greenville-New Bern-Washington	0.1543	0.0143	8.91	130

### Optimal refueling strategy

We now present the results of the optimal refueling control algorithm for the SIR deterministic model formulated in the methods section. We utilize the constant values of transmission rate per capita (β) and the recovery rate (γ) estimated from the procedure outlined in the previous section. The continuous time invariant SIR data from these parameters closely resemble the empirical data and can be utilized directly in the optimal control algorithm. The results for the optimal refueling strategy are in the form of per capita rate of refueling (u_v,max_) and the corresponding switching time (t_s_), which control the fuel shortage epidemic, i.e. lower the basic reproduction number (R_0_), to non-epidemic levels as presented in *Eq 27*. The per capita rate of refueling (u_v,max_) corresponds to the fraction of susceptible fuel stations, S(t), that will be provided with extra refueling scheme at a given time. We vary this refueling rate (u_v,max_) from 0 (no intervention) to 0.75 (75% refueling stations prevented from emptying), and determine the corresponding switching time (t_s_) for the intervention to effectively reduce the fuel shortage below epidemic levels. When viewed in totality, this analysis would provide a strategy to allocate limited resources to different affected regions from a hurricane.

**Fig 6:**
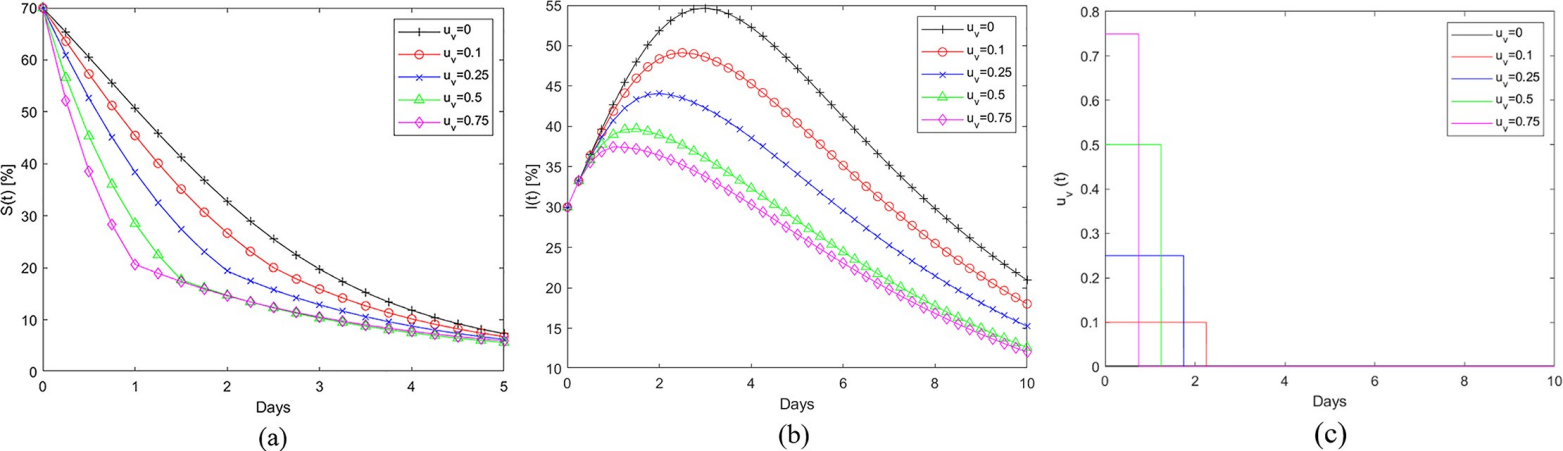
(a) Evolution of susceptible (operational) gas stations and the effect of refueling for Fort-Myers-Naples during Hurricane Irma. (b) Corresponding evolution of Infected or empty fuel stations. (c) The optimal application and switching time, t_s_, for different refueling rates.

**Fig 7:**
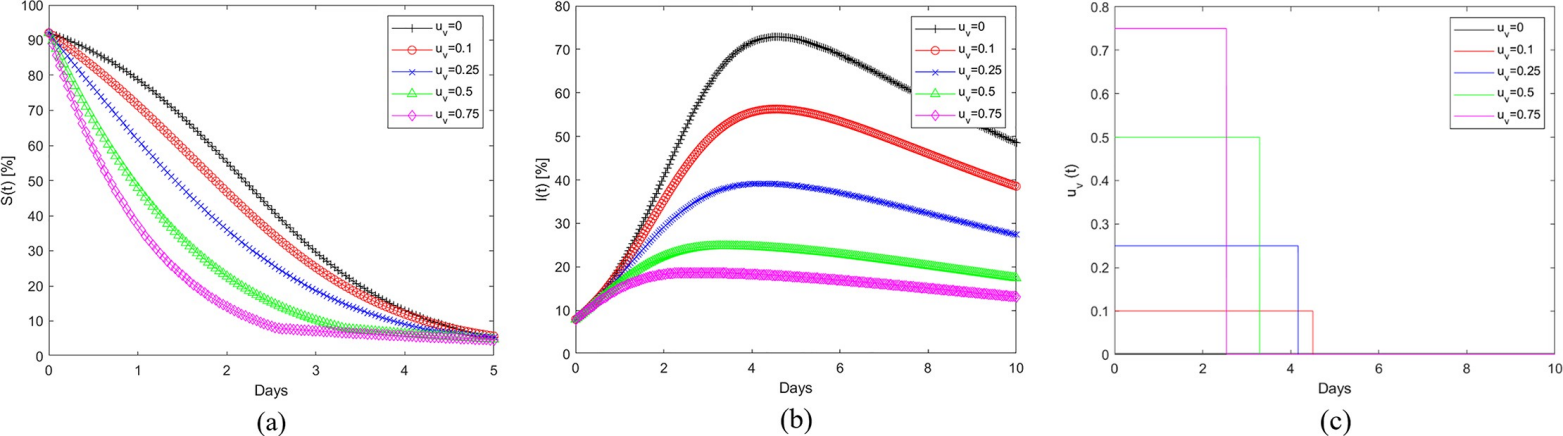
(a) Evolution of susceptible (operational) gas stations and the effect of refueling for Wilmington during Hurricane Florence. (b) Corresponding evolution of Infected or empty fuel stations. (c) The optimal application and switching time, t_s_, for different refueling rates.

**Fig 8:**
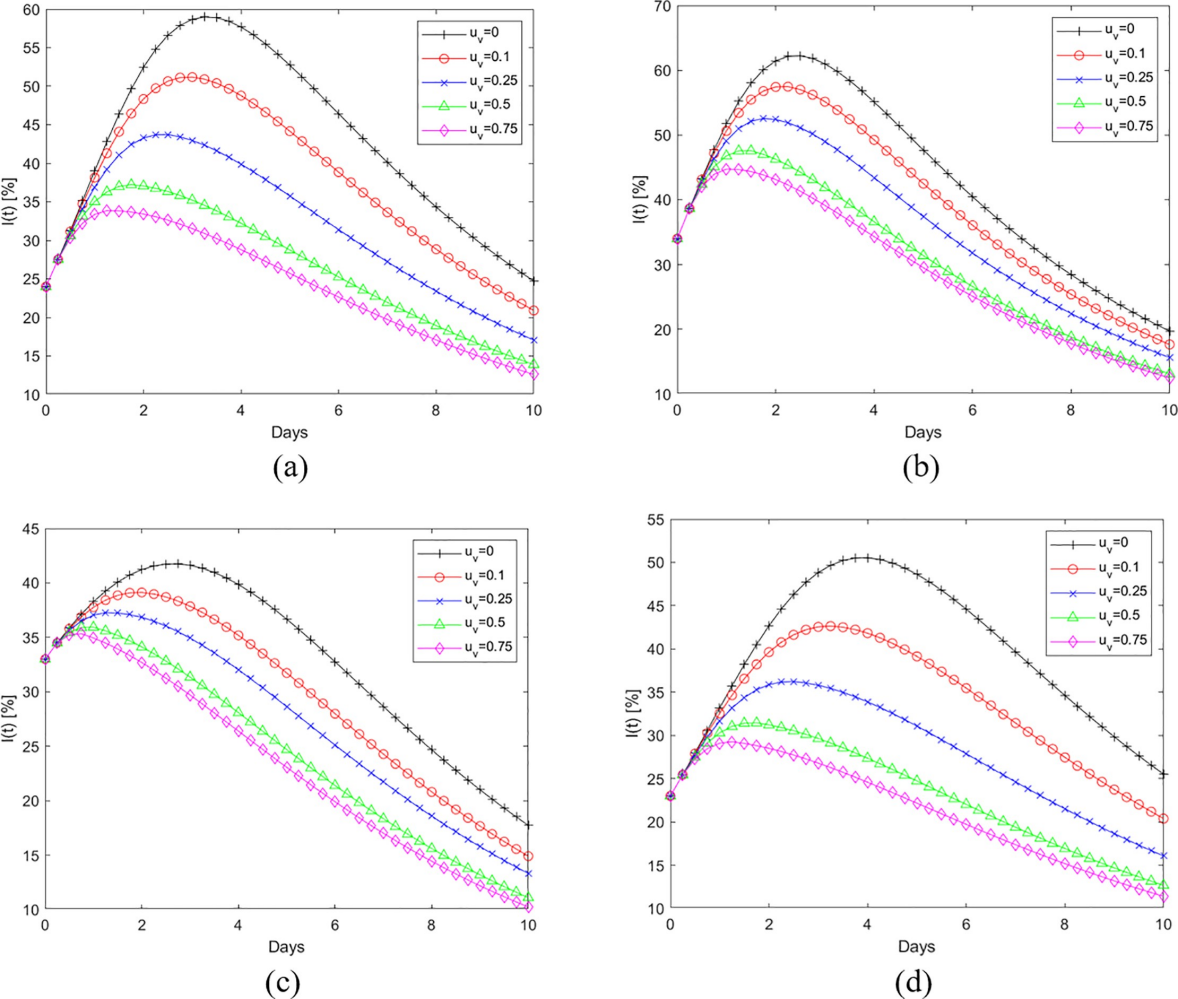
The evolution of empty gas stations and the effect of optimal refueling strategy on other cities affected by Hurricane Irma (a) Miami-Ft Lauderdale, (b) Tampa St Petersburg, (c) Orlando and (d) Jacksonville.

Fig 6(A), 6(B) and 6(C) show the application of the refueling strategy to the Fort Myers-Naples region during Hurricane Irma. Fig 6(A) shows the percentage fuel stations that remain operational at any given time. The baseline is the curve corresponding to u_v_ = 0 and is same as that in Fig 3(B). The baseline data generated using the UKF estimation process is the continuous time invariant representation of the empirical data and can be characterized by Eqs 1 and 2. The remaining plots in 6(a) correspond to different refueling interventions. In these instances, the per capita rate of refueling (u_v,max_), represents the fraction of gas stations that are prevented from becoming empty through external intervention till the switching time. The level of external intervention in terms of amount of fuel required changes every time step as the number of operational fuel stations (S(t)) changes. The application of this control strategy helps reduce the number of empty fuel stations, I(t), as shown in Fig 6(B). Fig 6(C) shows the application time and the switching time for the intervention. Here the per capita rate of refueling (u_v,max_) is applied from the beginning of the observed time window and then switched to zero at the time designated by the condition in Eq 27. Note that application period for the refueling is well in advance of the hurricane landfall (Day 4 in this case). Fig 6(B) and 6(C) show that the per capita refueling rate of 0.1 for 2.2 days reduces the peak fuel shortage from 55% to 48% and also moves the occurrence of peak shortage back by a day. When the u_v,max_ = 0.75 is applied, the application period required is 0.5 days and it reduces the peak shortage to 37%. Fig 7(A), 7(B) and 7(C) show similar data for Wilmington affected by Hurricane Florence. Similar trends for the effect of refueling strategy in reducing the susceptible gas stations and in pulling back the peak fuel shortage time can be observed here as well. Table 3 tabulates the switching times corresponding to different per-capita fueling rates for the cities affected by Hurricanes Irma and Florence. Fig 8 shows the evolution of infected or empty gas stations for other cities affected by Hurricane Irma, Miami-Ft Lauderdale, Tampa-St Petersburg, Orlando and Jacksonville. The β and γ values used to generate the baseline continuous time invariant SIR data corresponding to u_v_ = 0 are shown in Table 2. The reduction in fuel shortages with different levels of intervention u_v,max_ follows the same trend as that discussed earlier.

**Table 3:** Switching times (*t_s_*) corresponding to different per-capita refueling rates (*u_v_*) for the major cities affected by Hurricanes Irma and Florence.

Hurricane	City/Area	Switching time (t*_s_*) [Days] for			
		u*_v_* = 0.1	u*_v_* = 0.25	u*_v_* = 0.50	u*_v_* = 0.75
Irma	Miami-Ft Lauderdale	2.25	1.75	1	0.75
	Ft Myers-Naples	2	1.5	1.25	0.75
	Tampa-St Petersburg	2.25	1.75	1.25	0.75
	Orlando	2.75	2.25	1.50	1
	Jacksonville	1.75	1	0.75	0.5
Florence	Wilmington	3	2.25	1.5	1
	Greenville-NewBern- Washington	2.25	1.75	1	0.75

**Fig 9:**
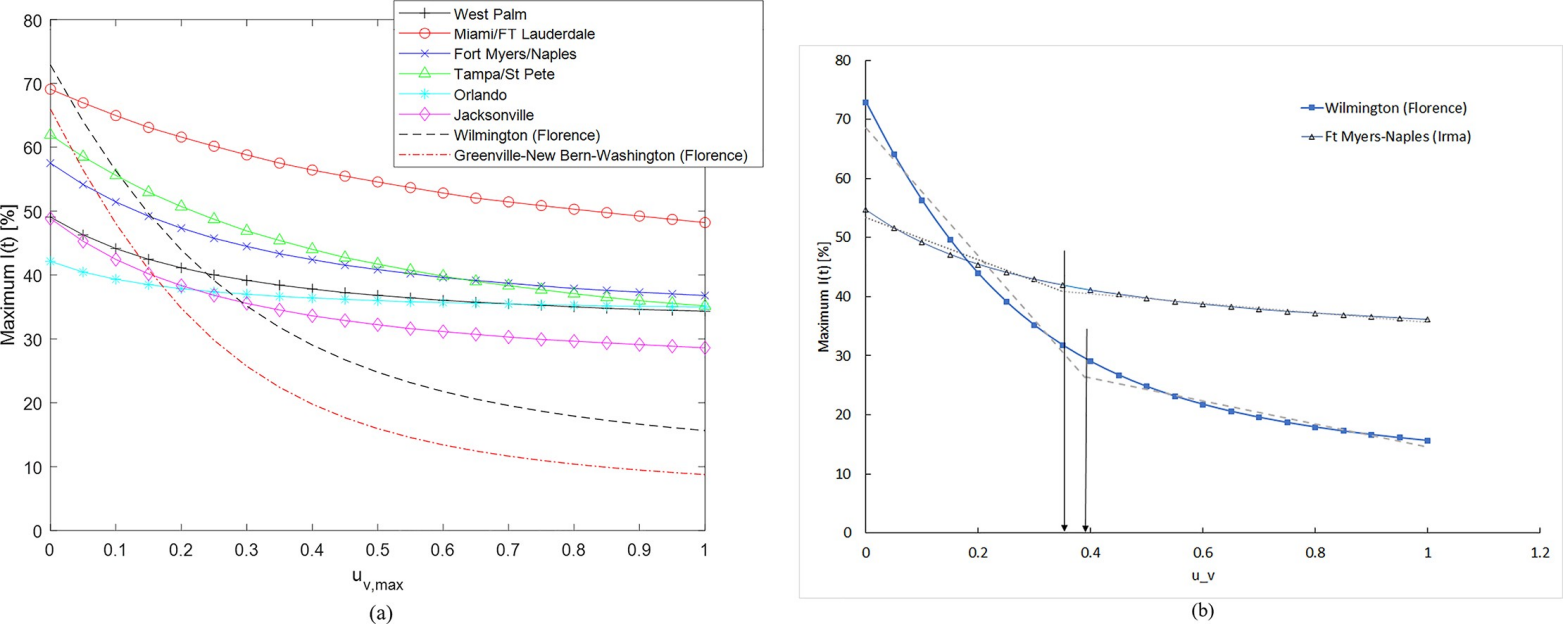
(a). Maximum number of empty fuel stations, I(t), for u_v,max_ ranging from 0 to 1. (b). Bilinear Interpolation of Wilmington and Ft Myers-Naples to determine the optimal u_v,max_. during Hurricanes Florence and Irma.

While increasing the refueling intervention levels reduces the number of empty refueling stations, there is a diminishing return when the intervention is increased beyond a certain level. Fig 9(A) shows the variation in the peak value of empty fuel stations (I(t)) as the refueling rate (u_v,max_) is increased from 0 to 1. The change of maximum I(t) is observed to be more in areas with higher rate of fuel shortage, i.e. high transmission rate per capita (β). For the Fort Myers-Naples area, with β = 0.0089/day there is a gradual reduction in maximum I(t) as u_v,max_ is increased. In the case of Wilmington, with β = 0.012/day, there is a steeper change in maximum I(t) at low u_v,max._ In all the cases, there is a steady decrease in I(t) with the increase in the refueling rate; however, the rate of decrease is clearly higher for lower values of u_v,max_. When this behavior is plotted as a bilinear relation as sown in Fig 9(B), the inflection point corresponds to the most effective refueling rate when there is a resource constraint.

An ongoing evacuation during a hurricane can be an evolving and dynamic problem. Residents in affected areas decide on the evacuation and preparation based on various factors like hurricane intensity, hurricane path and personal resources [42]. The ensuing fuel shortages can vary by location based on these factors. The approach presented here can help decision makers with resource allocation during an ongoing emergency. In an ongoing evacuation scenario, the UKF parameter estimation can be used to estimate the β parameter for the affected regions even with limited data during the beginning of the fuel shortage. The γ parameter is related to recovery rate and can be estimated using the β estimate and an approximate recovery period based on historical data. This analysis for all the affected regions combined with the optimal refueling methodology discussed above can help assess the levels of fuel supply required to mitigate the fuel shortage crisis in the affected regions, and thereby assist decision makers in allocating limited resources in a dynamically evolving emergency.

## Conclusions

Fuel is a critical and limited resource during a natural disaster. Regional evacuations from major hurricanes can generate significant and often overwhelming fuel demand. In this study, for the first time we utilize a combination of social media crowdsourced data and epidemiological modeling techniques to address the problem of fuel shortages during hurricanes. We used the crowdsourced data from Gasbuddy to model the fuel shortage experienced during recent hurricanes as a contagion. The Unscented Kalman Filter was utilized to evaluate the dynamic parameters, transmission rate per capita (β) and recovery rate (γ), and reproduction number (R_0_) for multiple cities affected by Hurricanes Irma and Florence. For example, we find that the R_0_ value for Miami- Fort Lauderdale area affected by hurricane Irma is 3.98. Further, An optimal refueling strategy was developed using Bang-Bang Control theory. The optimal control strategy provides useful insight into the control of a fuel shortage contagion using a vaccination analogue to the SIR model. Our results show that for Naples- Fort Myers affected by Hurricane Irma, a per capita refueling rate of 0.1 for 2.2 days would have reduced the peak fuel shortage from 55% to 48% and a refueling rate of 0.75 for half a day before landfall would have reduced to 37%. This approach can be used to analyze fuel shortages during an ongoing evacuation and assist in resource allocation decisions.

## Supporting information

S1 Data Supplemental Files include: (1) PE.m parameter estimation matlab code, (2) bangbangcontrol.m, optimal control matlab code, (3) Parameter estimation data–Irma_FlorenceData.xls, (4) Optimal control data for (a) Miami–MiA_OC_Data.xls, (b) Tampa- TPA_OC_Data.xls, (c) Fort Myers- Naples FTM_OC_Data.xls, (d) Jacksonville–Jax_OC_Data.xls, (e) Orlando–MCO_OC_Data.xls (f) Wilmington–WILM_OC_Data.xls, and (g) Greensboro–GNW_OC_Data.xls.
(ZIP)
